# State of nutrient management infrastructure of lagoons wastewater systems serving small communities in the U.S

**DOI:** 10.1088/3033-4942/ae5e00

**Published:** 2026-06

**Authors:** Denis Ruto, Ziya Jang, Pablo K Cornejo, Harold Leverenz, Kevin Orner

**Affiliations:** 1 Department of Civil and Environmental Engineering, West Virginia University, Morgantown, WV, United States of America; 2 Department of Civil and Environmental Engineering, University of California, Davis, CA, United States of America; 3 Department of Civil and Environmental Engineering, California State University, Chico, CA, United States of America

**Keywords:** stabilization ponds, nitrogen, phosphorus, carbon footprint, eutrophication, life cycle assessment

## Abstract

Lagoon wastewater treatment systems remain the backbone of sanitation in many small U.S. communities, yet their treatment technologies and nutrient management performance remain poorly characterized. Using publicly available federal and state datasets supplemented by permit reviews, this study evaluated the existing nutrient management infrastructure for municipal lagoon wastewater systems serving communities with less than 10 000 residents. Of all lagoon systems identified, approximately 69% of facilities operate at less than 0.1 million gallons per day. Non-aerated lagoons accounted for about 61% (2512 facilities), while aerated systems were present in 1697 facilities. Nutrient permitting was inconsistent throughout the U.S., where parameters such as total nitrogen (TN) and total phosphorus (TP) were frequently monitored but seldom subject to enforceable limits, constraining performance benchmarking and compliance comparability. Effluent data (2020–2024) showed moderate organics and suspended solids control but substantial variability in ammonia, TN, and TP, with many facilities exceeding limits typical of nutrient-sensitive watersheds. Facilities with nutrient-specific upgrades including moving bed biofilm reactors and fixed-bed attached growth reactors, consistently achieved low effluent ammonia and TN while chemical precipitation provided the most reliable TP reduction. Life cycle assessment of the operations phase indicated higher carbon and eutrophication footprints for facultative and aerated lagoons, whereas targeted nutrient management upgrades balanced nutrient removal and energy efficiency, lowering environmental impact profiles. The findings of this study underscore the need for improved influent data collection efforts and nutrient permitting as a driver for compliance. Priorities for practice include leveraging routine monitoring datasets to bolster compliance oversight and integrating decision-making frameworks.

## 1. Introduction

Lagoon wastewater treatment systems are among the most widely used treatment technologies in small U.S. communities, valued for their low capital and operating costs, simple maintenance requirements, and minimal energy demands [1]. These systems use natural biological processes, such as microbial activity and sedimentation in shallow basins, to treat domestic wastewater [2]. Their simplicity and adaptability make them especially attractive for rural areas with limited technical capacity and financial resources [3]. According to the U.S. Environmental Protection Agency (U.S. EPA), more than 8000 lagoon systems are currently operational across the United States, including discharging, non-discharging, and land-application systems [4].

Most lagoon systems were originally designed to meet secondary treatment standards, focusing on the removal of biochemical oxygen demand (BOD) and total suspended solids (TSS) [5]. As environmental regulations evolve to address nutrient pollution more stringently, many small communities now face the challenge of upgrading or modifying their existing lagoon systems to comply with total nitrogen (TN), ammonia-nitrogen (NH_3_–N), and total phosphorus (TP) limits [6]. This shift is driven by growing recognition of the impacts that excess nutrients can have on ecosystems and human health. Ammonia is acutely toxic to fish and other aquatic life at relatively low concentrations, while nitrate contamination in groundwater poses serious risks to drinking water safety, particularly for infants and vulnerable populations. Phosphorus and nitrogen also drive eutrophication in surface waters, resulting in algal blooms, hypoxia, and habitat degradation [1, 7, 8].

At a national scale, nutrient challenges in lagoon systems align with broader engineering priorities. Among the 14 grand challenges for engineering identified by the U.S. National Academy of Engineering, two are directly relevant to the issue of nutrient management in lagoon systems: *managing the nitrogen cycle* and *providing access to clean water* [9]. Inadequately treated wastewater is one of the major pathways through which nitrogen and phosphorus are released into the environment [10]. Consequently, improving nutrient removal and expanding opportunities for safe effluent reuse are not only operational needs for lagoon communities but also key national objectives within sustainable water management.

However, many small communities struggle to meet nutrient limits due to aging infrastructure, technical and managerial capacity issues, and financial constraints [11]. Upgrading lagoon systems or installing advanced nutrient removal technologies is often cost-prohibitive, particularly for communities with a limited tax base or those located in economically disadvantaged or geographically isolated areas [12, 13]. In response to these challenges, the U.S. EPA introduced the *Lagoon Wastewater Treatment Action Plan (2022–2026)* to support small, rural, and tribal communities in modernizing lagoon treatment. The action plan aims to ensure more equitable access to technical and financial resources and prioritizes improvements in nutrient removal performance to protect both public health and water quality [4].

Accordingly, the overall goal of this study was to assess the current state of lagoon wastewater systems in small U.S. communities, with a focus on nutrient management capabilities. Publicly available data from the U.S. EPA, clean watersheds needs survey (CWNS), and supplemental permit inventories were compiled to characterize system configurations, nutrient removal practices, and regulatory compliance. Despite their prevalence, lagoon systems remain poorly represented in national datasets, with gaps in influent data, performance reporting, and process details [4, 13]. This lack of visibility limits the ability of states and utilities to benchmark nutrient removal performance, allocate resources, and prioritize lagoon upgrades [14].

To address these gaps, this study pursues three objectives: (1) characterize existing lagoon infrastructure and associated environmental impacts, (2) evaluate current nutrient management performance, and (3) identify opportunities for feasible nutrient upgrading. By addressing the longstanding lack of visibility surrounding lagoon infrastructure and performance, this work aims to strengthen national efforts to reduce nutrient pollution, supports data-driven decision-making for lagoon-dependent communities, and informs state and federal programs seeking equitable investment in rural wastewater infrastructure. By systematically characterizing lagoon infrastructure, nutrient performance, and technology gaps at the national scale, this work provides the empirical foundation necessary to support evidence-based permitting, prioritize infrastructure funding, and guide the development of realistic nutrient management strategies. The findings therefore advance both the scientific understanding of decentralized treatment systems and their practical application in policy, permitting, and rural infrastructure planning.

## 2. Methods

Data for this study were compiled from a diverse range of publicly available sources, federal and state agency databases, vendor case studies, and expert outreach. The primary objective was to identify and characterize lagoon wastewater treatment facilities across the United States, with a particular focus on those serving populations under 10 000 or with design flows less than 1 million gallons per day (MGD).

Key federal data sources included the U.S. EPA’s enforcement and compliance history online (ECHO), the integrated compliance information system–national pollutant discharge elimination system (NPDES) database, and the CWNS for the years 2000 through 2022 [15–17]. Additional U.S. EPA resources consulted included the water pollution search tool, wastewater technology clearinghouse, and the 2022 lagoon inventory (a composite dataset compiled from 18 federal and state databases) [4, 18, 19]. Consultations with U.S. EPA staff involved in the lagoon inventory initiative provided further clarification on facility classification criteria and methods used to integrate and validate the data. State-level data were obtained from publicly accessible wastewater databases managed by state environmental agencies. These sources provided supplemental facility-level information, particularly where federal data were incomplete or outdated. Compliance data were further enhanced by reviewing general and individual NPDES permits.

All data were processed and analyzed using R (v3.6), Python (v3) and Microsoft Excel 2024. Facility records were merged using unique identifiers such as NPDES ID, facility name, and geographic coordinates. To isolate lagoon wastewater systems, filtering criteria were applied, primarily keyword matching of facility names containing terms such as ‘lagoon,’ ‘stabilization,’ or ‘pond.’ A comprehensive data set was then developed to consolidate information on municipal lagoon systems from all utilized data sources. For verification and quality assurance, facilities were cross validated using permit reviews from ECHO, satellite imagery, and visual confirmation of lagoon cell counts. No formal misclassification rate was calculated since lagoon identification relied on multiple independent validation steps to reduce false positives. Nevertheless, some degree of misclassification, false exclusion or inclusion cannot be ruled out was due to variability in facility naming conventions and reporting practices. The resulting integrated dataset formed the foundation for subsequent analyses of system characteristics, regulatory performance, and contextual suitability.

Vendor case studies and expert outreach were used qualitatively to support technology characterization, performance ranges, and operational considerations rather than to define the facility cohort or inform quantitative analyses. Vendor materials were screened for relevance to lagoon applications and, where possible, cross-referenced with peer-reviewed literature to reduce the potential for promotional bias. Expert outreach was conducted to contextualize observed patterns and identify commonly reported practices across lagoon systems, rather than to highlight exemplary or unusually high-performing facilities. While these inputs enhanced interpretation of technology options, their use may have introduced some degree of bias toward better-documented or more successful upgrade cases.

Life cycle assessment (LCA) was conducted for each facility following ISO 14 040 standard four-phase framework: (1) goal and scope definition, (2) life cycle inventory analysis, (3) impact assessment, and (4) interpretation [10, 20]. The system boundary encompassed the preliminary treatment, secondary treatment, and tertiary treatment processes, and was limited to the operational phase of the treatment system. Energy (e.g. electricity for aeration) and chemical such as chlorine and other inputs associated with routine operation were included, while solids handling and management processes were excluded from the boundary. Infrastructure construction, end-of-life decommissioning and upstream collection systems were also excluded.

The operations phase was emphasized because prior work showed that operational activities are the dominant contributors to life-cycle environmental impacts in lagoon systems [21]. The functional unit for the analysis was one cubic meter of treated wastewater. Where facility-specific influent concentration data was unavailable, influent mass loading was estimated using average per-capita loading rates reported in peer-reviewed literature, scaled by the population served at each facility [1, 22]. These estimates were used to support comparative assessment of loading and treatment performance rather than precise facility-level quantification.

The LCA focused on three key indicators: carbon footprint, freshwater eutrophication potential (FEP), and marine eutrophication potential (MEP), representing carbon and nutrient management challenges, respectively. The carbon footprint was estimated using influent BOD as a proxy for organic loading, applying methane (CH_4_) and nitrous oxide (N_2_O) emission factors from the 2019 IPCC guidelines and expressed as kilograms of CO_2_ equivalent using a 100 year time horizon [23]. Indirect emissions associated with electricity consumption were incorporated using regional fuel mixes (coal, oil, natural gas, hydro, nuclear, wind, solar, biomass, geothermal, and other purchased fuels) and corresponding emission factors derived from SimaPro v9.5 (Ecoinvent 3 and USLCI databases, and TRACI 2.1 methodology)[24]. Embodied emissions from alum use were included for facilities employing chemical phosphorus removal, based on an average dose of 0.03 kg m^−3^. Embodied carbon associated with infrastructure (construction phase) was excluded from this analysis because facility-specific material inventories and construction specifications were not available for the study cohort. Construction-phase contributions to life-cycle impacts are known to be context-dependent and, in some lagoon system assessments, have been reported to represent a relatively smaller share of total impacts compared to operational emissions [25, 26].

FEP and MEPs were calculated using reported effluent concentrations of TP, TN, and nitrate (NO_3_–N), which were converted to nutrient-equivalent loads using facility design flows. Characterization factors from TRACI 2.2 were applied for both FEP (kg P eq mm^−3^), and MEP (kg N eq mm^−3^), because of its suitability for U.S. contexts and county-level spatial resolution [27]. Indirect contributions from chemical production and electricity generation were incorporated for both FEP and MEP using emission derived from SimaPro v9.5 (Ecoinvent 3 and USLCI databases, and TRACI 2.1 methodology). All impacts were calculated per cubic meter of treated wastewater to ensure comparability across the lagoon facilities. Infrastructure lifetime was assumed to be 25 years consistent with commonly reported design lives for lagoon treatment components [11, 28].

Key modeling assumptions with strong influence on LCA outcomes included influent characteristics, energy consumption and chemical inputs. Influent rates were based on reported design flows for each facility. Mass loadings were estimated based on the population served reported for each facility and common per capita ranges in the U.S. reported in peer-reviewed literature [1, 22]. Due to limited availability of site-specific operational data for small community lagoons, proxy variables were used to represent energy demand, chemical consumption, and material quantities. These proxies were derived from a synthesis of peer-reviewed literature, vendor technical documentation, and engineering design guidance. While this approach introduced uncertainty, this approach is consistent with prior comparative LCAs of small community wastewater treatment technologies and is appropriate for early-stage screening analyses where detailed facility data are unavailable [26, 29].

## 3. Results and discussion

### 3.1. Influent characteristics

**Figure 1.**
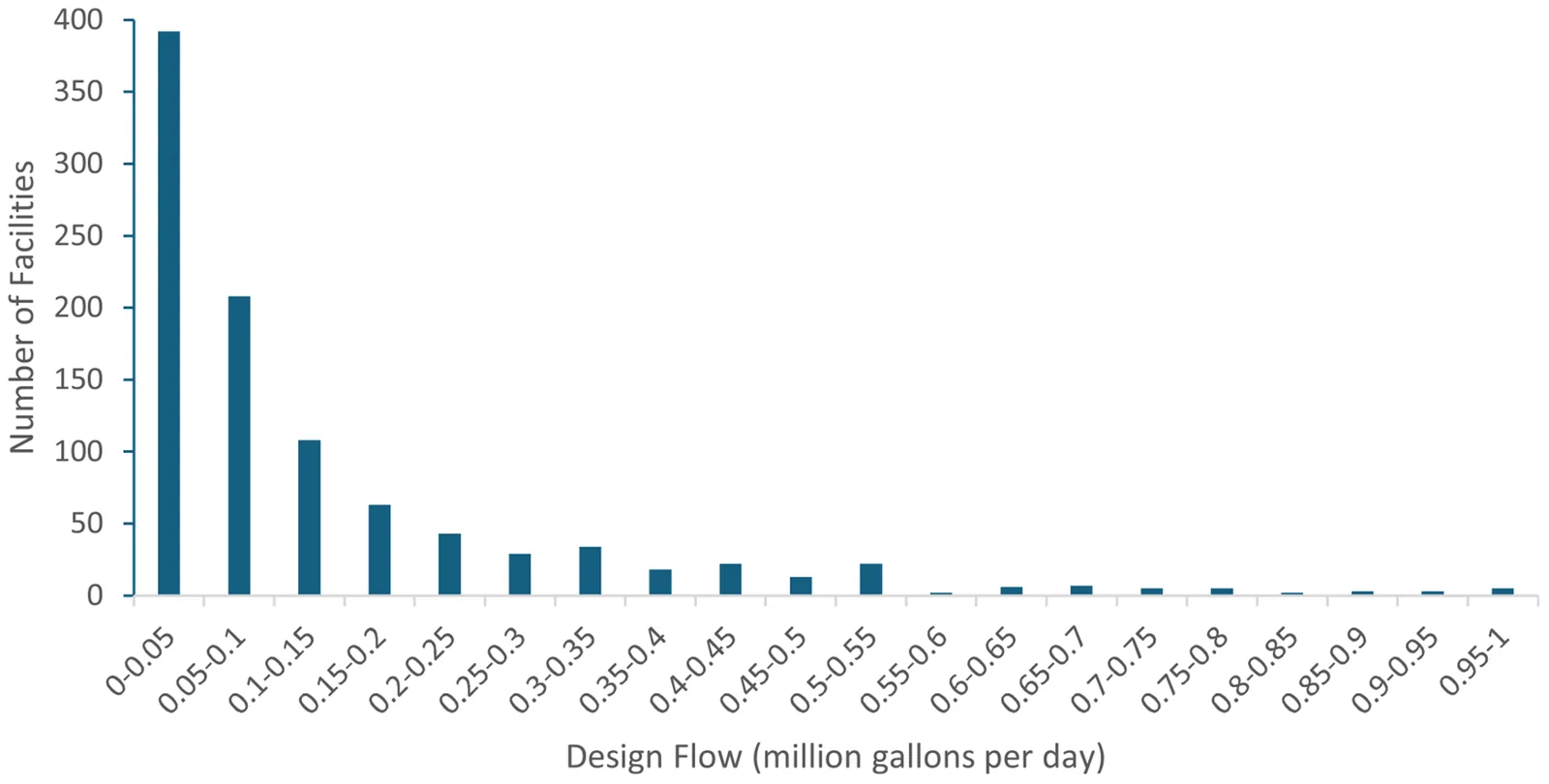
Distribution of discharging lagoon wastewater systems based on design flow rate (million gallons per day).

Influent flow rates across the identified lagoon systems varied widely, reflecting the diversity in system size and service population. The mean flow was 0.138 MGD with a standard deviation of 0.175 MGD, indicating high variability in design flow (figure 1). About 45% of facilities (392 systems) operated at flows below 0.05 MGD, emphasizing the predominance of very small-scale lagoon systems. When extended to facilities operating under 0.1 MGD, this number jumps to 600 facilities, or approximately 69% of the total. Flow categories above 0.25 MGD represent only a small fraction of the dataset, with each subsequent bin contributing progressively fewer facilities.

Synthesized influent wastewater data showed moderate to high concentrations of organic matter and nutrients (table 1). Organic loading BOD and cBOD was high, with mean concentrations of 161.3 mg l^−1^ and TSS at 147 mg l^−1^, both peaking above 400 mg l^−1^ at the 95th percentile. Influent nutrient concentrations varied significantly. Ammonia averaged 25.2 mg l^−1^, ranging from 1.4 to 54.4 mg l^−1^. Total kjeldahl nitrogen (TKN) and TN were relatively high, averaging 34.9 mg l^−1^ and 36.0 mg l^−1^, respectively. Meanwhile, nitrite and nitrate (NO_2_^−^ + NO_3_^−^) remained low (mean: 2.2 mg l^−1^), consistent with minimal nitrification occurring prior to lagoon treatment [30]. TP concentrations averaged 5.01 mg l^−1^, as expected in municipal wastewater [7].

Evaluating influent characteristics proved challenging, as these data are often missing since they are not consistently required under the NPDES program. Unlike effluent monitoring, influent sampling is often absent from permit conditions. Thus, compiling representative values required mining data from individual NPDES permits, historical engineering reports, and past submissions, many of which are not standardized or centrally available. This limitation underscores a broader gap in regulatory data collection, particularly for small community lagoon systems.

### 3.2. Existing treatment processes

#### 3.2.1. Pre-treatment

Pre-treatment is a critical component of lagoon wastewater treatment systems, serving to remove solids, grit, and other coarse materials that could otherwise impair lagoon performance or reduce the effective treatment volume [5]. In the reviewed dataset, screening emerged as the most widely used pre-treatment unit, recorded in at least 98 facilities. Other pre-treatment methods reported included grit removal (21 facilities), comminution (17 facilities), equalization (6 facilities), Imhoff tanks (3 facilities), and pre-treatment aeration (2 facilities). Most lagoon systems employed simple pre-treatment trains, often relying on a single unit process. Screening alone was the most common configuration (72 facilities), while combinations like screening followed by grit removal (11 facilities), comminution alone (7 facilities), and comminution with screening (5 facilities) reflect low-cost setups typical of small communities.

While these results may not fully represent the actual pretreatment infrastructure present in lagoons, owing to limitations in the reported dataset and permit information, they nevertheless provide a useful starting point for retrofitting lagoons where pretreatment is lacking. Such retrofits are important for protecting downstream processes from clogging, sediment accumulation, and hydraulic short-circuiting [2]. Effective pre-treatment thus forms the first line of defense in nutrient management, ensuring subsequent lagoon processes operate under optimal conditions.

#### 3.2.2. Secondary treatment

**Figure 2.**
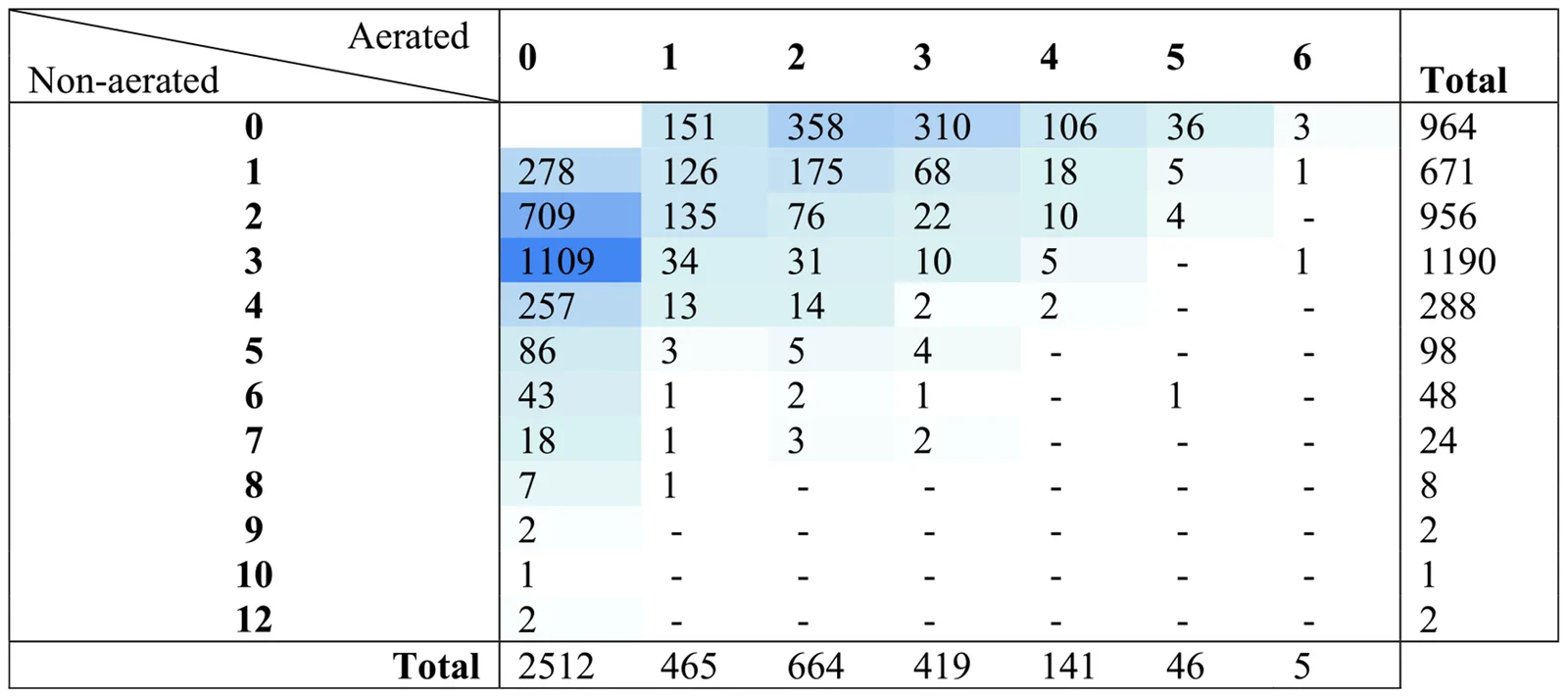
Distribution of the number of cells in aerated and non-aerated lagoons. Darker shade represents the most prevalent type.

Among lagoon systems for which cell configuration information was available, 1697 lagoon systems were identified as aerated and 2652 as non-aerated. The configuration of lagoon systems varied widely in terms of the number of aerated and non-aerated cells, as shown in figure 2. The most common configuration, found in 1109 systems, consists of three non-aerated cells in series. Two other frequent configurations include systems with two non-aerated cells (709 systems) and one non-aerated cell (278 systems), commonly facultative ponds coupled to a maturation pond.

Approximately 61% of identified lagoon facilities operated with non-aerated cells, highlighting the ongoing dominance of traditional passive lagoon systems in small communities. These systems typically consist of a series of facultative and maturation ponds, with the first one or two cells functioning as facultative ponds that support both anaerobic and aerobic microbial processes in the anoxic and anaerobic layers [2]. The downstream cells, often maturation ponds, are shallower and promote additional pathogen reduction and limited nutrient attenuation through extended retention time and natural sunlight exposure [5].

While passive systems are valued for their simplicity, they have limited capacity for ammonia removal and nutrient reduction, especially under cold weather or high loading conditions [30]. This limitation has driven the integration of aerated cells into an increasing number of lagoon systems (1697). Aerated lagoons enhance oxygen transfer, allowing for more effective BOD removal and facilitating nitrification to convert ammonia into nitrates [31]. These systems are especially valuable as front-end treatment units where the influent requires rapid stabilization [12].

Many aerated systems reflected hybrid treatment trains that couple mechanically aerated cells in the early stages with the extended detention and polishing capacity of maturation ponds downstream. Examples of common configurations included 2 aerated cells (358 systems) and 2 aerated + 1 non-aerated cells (175 systems), with the latter demonstrating a possible retrofit of enhancing traditional designs rather than replacing them entirely. This hybrid approach would allow communities to meet tighter regulatory limits, particularly for ammonia, without abandoning the cost-efficiency and low sludge generation benefits of facultative systems [30]. More complex configurations such as those with five or more cells or more than three aerated cells were relatively rare, typically associated with larger service populations, advanced effluent requirements, or facility upgrades [32].

**Figure 3.**
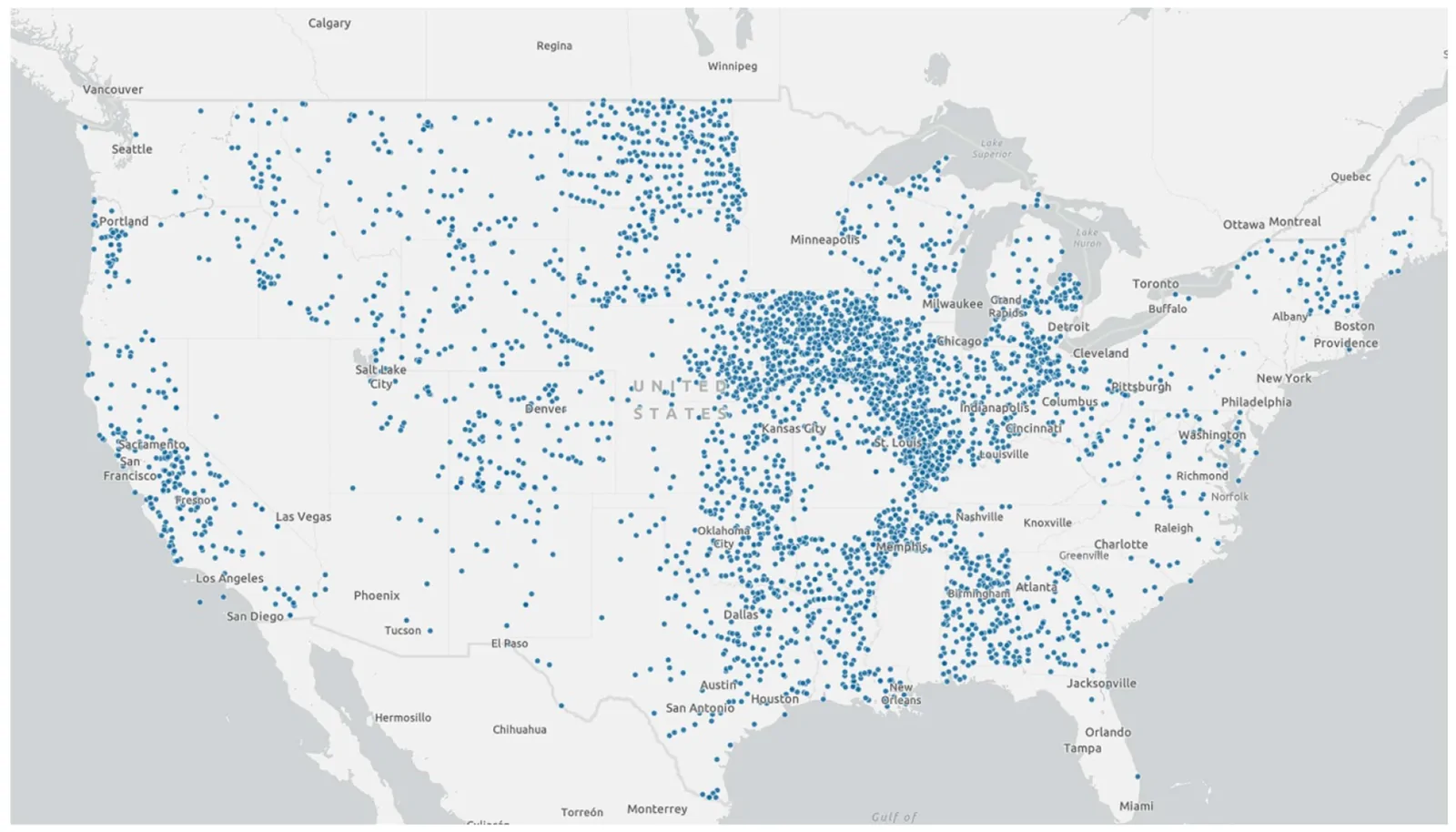
Spatial distribution of lagoon wastewater treatment systems (<1MGD) across contiguous 48 states in the U.S. (*N* = 4678).

The geographic distribution of lagoon system types reflected these design trends (figure 3). Non-aerated lagoons dominated in states with large rural populations or in regions where passive treatment is preferred for cost effectiveness and low operational requirements. North Dakota (304 non-aerated systems), Iowa (404), Kansas (300), and Minnesota (124) illustrated this reliance on passive systems. Nebraska, for instance, had 116 non-aerated systems compared to just 4 aerated based on our data, possibly signaling a preference to low-energy treatment options.

Conversely, aerated lagoons were more prevalent in states facing stricter discharge regulations or colder climates that limit the effectiveness of unaerated systems. Illinois (257 aerated systems), Iowa (310), Missouri (124), and Oklahoma (88) are notable examples. States like Colorado (77), Texas (65), and Alabama (51) also reported widespread use of aerated systems, often integrated into hybrid treatment trains. States such as Missouri (124 aerated, 140 non-aerated), Montana (43 aerated, 46 non-aerated), and Texas (65 aerated, 90 non-aerated) showed relatively balanced adoption of both technologies, suggesting that treatment decisions were by and large driven by site-specific conditions, notwithstanding state design standards, funding availability, and permitting constraints.

#### 3.2.3. Lagoon modifications

Lagoon modifications are often implemented to improve treatment performance, manage discharge timing, or adapt systems to specific environmental contexts [1]. Among the internal features used to enhance lagoon performance, baffles were by far the most widely adopted, present in 202 systems. Baffles play a critical role in improving hydraulic retention time and minimizing short-circuiting, thereby enhancing treatment efficiency in lagoon cells [33].

Although lagoon recirculation is noted in literature as a viable upgrade alternative, there were only two reported instances from the data reviewed. Notwithstanding the data limitation, this may be indicative that lagoon recirculation in small community lagoon systems remains rare, possibly due to the added cost and complexity associated with implementing recirculation in small or resource-limited communities [34].

Controlled discharge featured in 24 facilities, whereby regulated effluent release was based on storage capacity and environmental conditions. A related approach, known as hydrograph-controlled release, was identified in 6 systems. In this setup, effluent is released only when stream flow is high enough to protect water quality, with storage lagoons used to hold wastewater during low-flow periods [35]. Complete retention systems, where no discharge occurs, were reported in ten facilities, providing an alternative for areas with stringent effluent NPDES limitations or sensitive receiving waters [14]. It is important to note, however, that the NPDES dataset represents only a portion of lagoon systems; nearly over 4000 non-discharging lagoons not regulated under NPDES are most likely land-application systems, meaning the true prevalence of these techniques is substantially higher than reflected here [14].

Various land application techniques, such as slow-rate irrigation and overland flow, were each reported in only 6 systems within the NPDES dataset, reflecting a small but notable adoption. These methods apply wastewater to land surfaces where soils and vegetation provide treatment through filtration, adsorption, microbial activity, and plant uptake [5]. Beyond contaminant removal, they also support co-benefits such as groundwater recharge and agricultural reuse [36]. While relatively low in maintenance demands, their implementation can be limited by soil permeability and higher upfront costs [37].

#### 3.2.4. Post-treatment

Post-treatment processes in lagoon systems are typically designed to ensure that final effluent standards are met, with the focus placed on solids removal and disinfection rather than nutrient reduction. Among the facilities reviewed, rock filters (31 facilities), rapid sand filters (20 facilities), intermittent sand filters (70 facilities), and slow sand filters (60 facilities) were reported in use. These technologies are attractive for small communities because they provide effective polishing with relatively low energy requirements and operational complexity [1]. Less frequently implemented were microstrainers (3 facilities) and mixed media filters (2 facilities), suggesting limited adoption of more specialized or resource-intensive approaches.

Chlorination was the most used disinfection method, observed in 267 facilities. However, chlorinated effluents can introduce harmful byproducts into receiving waters [38]. As such, dechlorination is implemented to reduce this impact as featured in 69 systems which include dechlorination steps. Non-chemical disinfection alternatives, such as ultraviolet and radiation-based systems, were reported in 33 systems each, indicating a possible shift to environmentally safer options [36].

While disinfection dominates post-treatment design, relatively few systems incorporate processes that directly target nutrient polishing. Only a limited number of systems reported using polishing (25 facilities), sedimentation (10 facilities) or clarification (8 facilities) as additional post-treatment steps, which could aid in further nutrient removal. The scarcity of dedicated nutrient-focused post-treatment suggests a significant gap in addressing nutrient discharges from lagoon systems. This underscores the need for more integrated strategies that extend beyond pathogen removal to tackle persistent nitrogen and phosphorus pollution prior to final discharge or reuse.

### 3.3. Effluent standards

#### 3.3.1. Permits

In the United States, discharging lagoon systems are regulated under the NPDES, a program established by the clean water act to control point source pollution and protect surface water quality [39]. NPDES permits specify allowable pollutant discharge limits and outline monitoring and reporting requirements for regulated facilities. A key distinction within this framework lies between parameters that are ‘permitted’, those with numerical discharge limits that must be met to maintain compliance, and those that are ‘monitored’, which must be measured and reported but are not subject to enforceable limits [14]. When applied to lagoon systems, this distinction reveals a fragmented regulatory landscape in which some pollutants have stringent limits, while others are widely tracked but remain unregulated.

As shown in table 2, organics (BOD/cBOD) and suspended solids TSS were almost universally permitted across lagoon systems. Of the 3959 total facilities, 3842 are permitted for BOD/cBOD, and 3848 out of 3966 are permitted for TSS. These parameters also have the most extensive data records, with 619 639 and 586 135 data points, respectively. In contrast, nutrient parameters, despite their well-documented role in eutrophication and water quality degradation, were far less consistently regulated. TN, for instance, was monitored at 737 of 800 facilities but only permitted at 63, while TP is monitored at 1301 of 1518 facilities and permitted at just 217. The same trend appears across specific nitrogen species. Ammonia was relatively well covered, permitted at 1442 of 3035 facilities and monitored at 1593, reflecting a stronger regulatory presence due to its acute toxicity to aquatic life. However, TKN and nitrate/nitrite are permitted at just 36 and 26 facilities, respectively, despite being monitored at 577 and 683, respectively.

This widespread monitoring without corresponding discharge limits reflects a fragmented and inconsistent approach to nutrient management within the NPDES framework for lagoon systems. This may be due in part to the complexity and variability of nutrient dynamics in lagoon systems, as well as historical focus on organic loading rather than nutrient pollution [14]. Nevertheless, the vast nutrient datasets produced through routine monitoring provide an opportunity to reinforce compliance oversight. As more states move toward adopting numeric nutrient criteria and tightening discharge requirements, aligning monitoring efforts with enforceable limits under NPDES will be essential.

**Figure 4.**
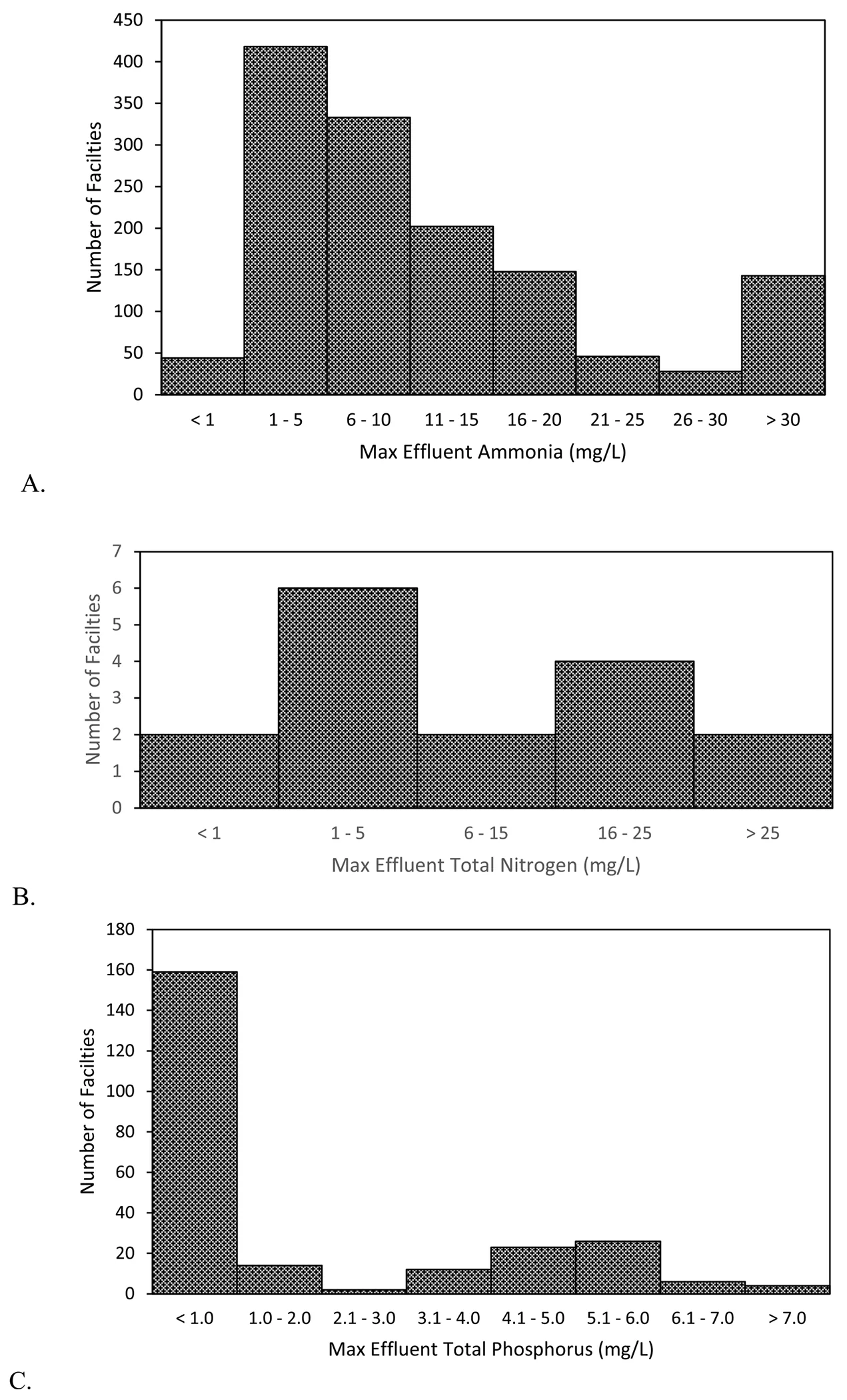
Maximum permit values for nutrient parameters in lagoon systems: (a) ammonia, (b) total nitrogen, and (c) total phosphorus. Distributions reveal wide variability and inconsistent regulatory thresholds across facilities.

Permit values where they do exist, especially for nutrients, vary widely (figure 4). For ammonia, which is more commonly regulated, maximum permitted concentrations range from below 1 mg l^−1^ to as high as 30 mg l^−1^. This variability reflects inconsistent state-level regulations and seasonal considerations. For TN, the few facilities with permits show a broad range between 5 and 25 mg l^−1^, while TP permit values are more clustered, with the majority of systems limited to 1 mg l^−1^. These distributions highlights the absence of national consistency in nutrient discharge standards for lagoon systems, even among those with active permits.

#### 3.3.2. Effluent quality

Analysis of effluent monitoring data from identified lagoon facilities for the years 2020–2024 revealed considerable variability in nutrient concentrations (table 3). This variability reflects the inherent treatment limitations of lagoon systems, particularly in achieving reliable nutrient removal [2]. Ammonia concentrations averaged 1.48 mg l^−1^ (geometric mean) with a standard deviation of 8.75. This suggests that while most systems achieved moderate ammonia removal, a notable fraction discharged at elevated concentrations. The 95th percentile value of 23.5 mg l^−1^ underscores the susceptibility of lagoons to operational and environmental constraints.

Elevated concentrations of TN and TP further underscore the nutrient management challenges facing lagoon-based systems. The median TN across 381 facilities is 8.7 mg l^−1^, while TP has a median of 1.9 mg l^−1^, both exceeding discharge limits typically applied in nutrient-sensitive watersheds (⩽5 mg l^−1^ TN; ⩽1 mg l^−1^ TP) [13]. It can be extrapolated from the data that most lagoon systems lack active nitrification–denitrification or enhanced phosphorus removal processes. These trends are reinforced by low nitrate/nitrite levels (median: 1.0 mg l^−1^), indicating limited nitrification.

**Figure 5.**
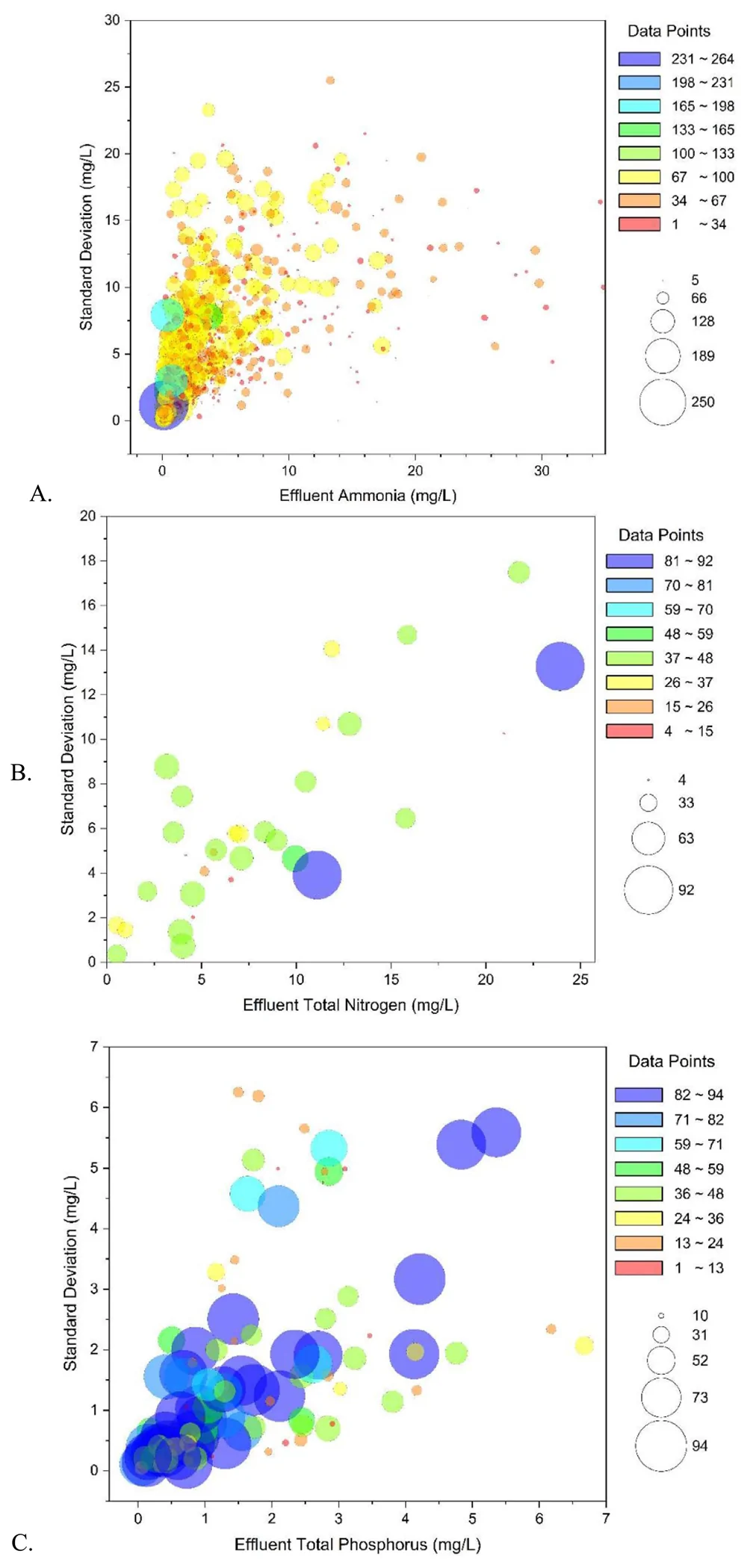
Distribution of compliance data for small community lagoons with ammonia, total nitrogen and total phosphorus permit limits.

Focusing on nutrient removal, there was wide spread of effluent ammonia concentrations, indicating substantial variability among facilities (figure 5). Although there were many facilities reporting elevated and inconsistent effluent ammonia (represented by the yellow and orange bubbles), there was a substantial number of lagoons that achieved both low effluent concentrations and high consistency (large blue and green clusters near the origin). This distribution highlights that while ammonia control is achievable, it remains highly site- and process-dependent.

TN had similar trends to ammonia, which showed significant variability in nitrogen removal efficiency. Systems with effluent TN below 10 mg l^−1^ generally showed low standard deviations, whereas those exceeding 15 mg l^−1^ exhibited greater variability. For TP, there was relatively low effluent concentration (<3 mg l^−1^) with small standard deviations, indicating higher consistency in phosphorus control compared to nitrogen. Most data points clustered below 2 mg l^−1^ with standard deviations under 2 mg l^−1^, reflecting the strong performance of P-removal systems.

It is important to note that facilities reporting TN and TP data represent a smaller and potentially non-random subset of the overall lagoon facility population, as these parameters are more commonly monitored at facilities subject to nutrient limits or enhanced regulatory oversight. Consequently, such facilities may exhibit systematic differences in treatment configuration or operation that are not representative of other lagoon systems. Accordingly, results for TN and TP may not be directly comparable to ammonia performance metrics derived from a broader reporting base and should be interpreted in this context.

### 3.4. Infrastructure capacity for nutrient management

#### 3.4.1. Nutrient management infrastructure

A wide range of supplemental unit processes have been implemented in small community lagoons for nutrient control, although their distribution across facilities is highly uneven (table 4). Aeration was the most common, having been identified in 1697 facilities. However, the specific configuration employed, such as partial-mix versus complete-mix systems or surface versus diffused aeration, could not be distinguished from the available data.

Baffles, featured in 202 facilities, minimize short-circuiting and enhance hydraulic efficiency, thereby indirectly improving nutrient removal [2]. Polishing units such as slow sand filters (60), rapid sand filters (20), and rock filters (31) were present in several facilities, primarily aiding in the removal of suspended solids and algae, thereby contributing to particulate nutrient reduction to some extent.

Chemical treatment is one of the most common approaches in removing phosphorus. This can be done either as batch or continuous overflow dosing of aluminum sulfate (alum) or ferric chloride [1, 13]. However, publicly available data showed limited application of chemical addiction in only 15 facilities. Notwithstanding the limited information available on small community lagoons, this limited adoption of chemical phosphorus removal may stem from minimal to no requirements on regulating phosphorus.

More recently, technologies designed specifically for nutrient removal have been implemented to further enhance lagoon performance. Notable examples include fixed-bed attached growth (FBAG) reactors (38 facilities), moving bed biofilm reactors (MBBRs) (23 facilities) and covered complete-mix lagoons with polishing (CCMP) (49 facilities). These advanced add-ons provide biological nutrient removal through mechanisms like enhanced nitrification and phosphorus uptake and represent a shift toward more innovative, modular upgrades capable of meeting stringent effluent limits.

Constructed wetlands (CWs) featured in 13 facilities while floating treatment wetlands (FTWs) were reported in only 2 facilities that used water hyacinth, indicating their relatively rare application. FTWs rely on buoyant platforms that allow emergent plants to establish in the wastewater lagoon, often too deep for natural rooting, with plant roots extending downward to form dense mats. In both CWs and FTWs, root network and platform surfaces provide habitat for biofilms, which play a key role in nutrient transformation alongside direct uptake by plants. Although CWs and FTWs can improve nutrient removal, their broader use is constrained by climate sensitivity and the labor required for plant harvesting [40, 41].

#### 3.4.2. Nutrient removal performance

**Figure 6.**
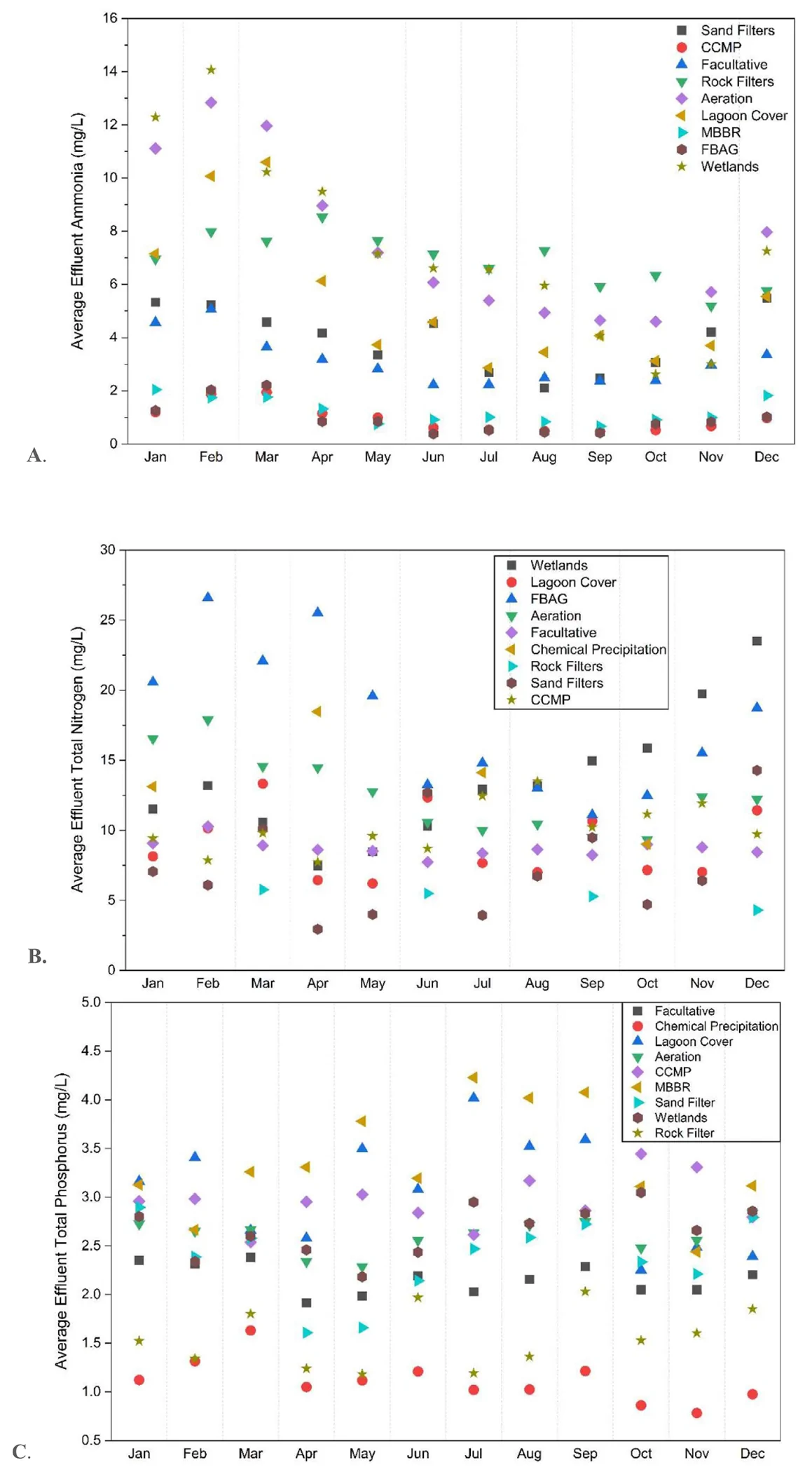
Performance of existing nutrient management technologies currently implemented in small community lagoons throughout the year. CCMP: covered complete-mix aerated lagoon with polishing; FBAG: fixed-bed attached-growth reactor; MBBR: moving-bed biofilm reactor.

Comparing the performance of existing nutrient management technologies indicated clear differences in nutrient removal efficiency and seasonality (figure 6). As shown figure 6(a), average effluent ammonia concentrations peaked during winter (January–March) due to limited microbial-led ammonia removal as a result of temperature drops, and limited operations from ice formation in some cases. This was observed particularly in passive systems such as facultative ponds, rock filters, and wetlands, which depend heavily on temperature-sensitive biological processes. With warmer temperatures in late spring and summer (May–September), there were lower effluent ammonia concentrations across all systems due to improved nitrification. Mechanically assisted technologies (MBBR, FBAG, and CCMP) exhibited the best performance, maintaining effluent ammonia levels below 2 mg l^−1^ year-round due to continuous aeration and biofilm-mediated nitrification that sustain microbial activity even under cold conditions.

Similar trends were observed for TN removal with respect to specialized nutrient removal upgrades (figure 6(b)). CCMP and MBBR maintained TN effluent concentrations below 10 mg l^−1^, while facultative lagoons and aerated systems show higher winter TN (>20 mg l^−1^). Wetlands demonstrated improved performance during the summer owing from vegetation growth.

On the other hand, phosphorus removal showed a less pronounced seasonal pattern but stronger dependence on treatment type (figure 6(c)). Chemical precipitation achieved the most stable and lowest effluent TP (~1.0–1.5 mg l^−1^) regardless of season, underscoring its high efficiency and low temperature sensitivity. Although there was no clear correlation of phosphorus transformations to temperature sensitivity in comparison to nitrogen pathways, seasonal changes affecting algal productivity, sediment–water interactions from thermal stratification, and oxygen availability, ultimately contribute seasonal fluctuations in effluent TP [13].

#### 3.4.3. Nutrient permit compliance

A closer look at the compliance of facilities with stringent nutrient limits revealed a wide range of nutrient management strategies (tables 5 and 6). Across the 993 facilities with reported ammonia limits, compliance with permit requirements ranged from 53% to 86%. These facilities featured several technologies including stand-alone facultative systems (48%), aeration (37% facilities), covered complete-mix lagoon with poling (CCMP) (25 facilities), moving-bed biofilm reactors (MBBRs) (12 facilities), fixed bed attached growth reactors (FBAGRs) (36 facilities), rock filters (17 facilities), sand filters (26 facilities) and CWs (12).

29 facilities were identified with TN limits with average compliance rate of 98%. Aeration was the most common technology implemented (17 facilities) with effluent TN geometric mean ranging from 0.5–21.8 mg l^−1^. FBAGRs featured in 6 facilities with effluent TN geometric mean ranging from 2–15.8 mg l^−1^. CW and MBBR featured in one facility each, with average effluent TN of 6.6 and 8.9 mg l^−1^, respectively.

With regards to TP, chemical precipitation was identified in 15 facilities, with geometric mean effluent TP ranging from 0.16 to 2.85 mg l^−1^. Facilities using aeration (*n* = 30) and facultative lagoons (*n* = 29) achieved 90% and 88% compliance with their respective TP limits, which are likely among the most stringent across lagoon systems.

### 3.5. Environmental impacts

**Figure 7.**
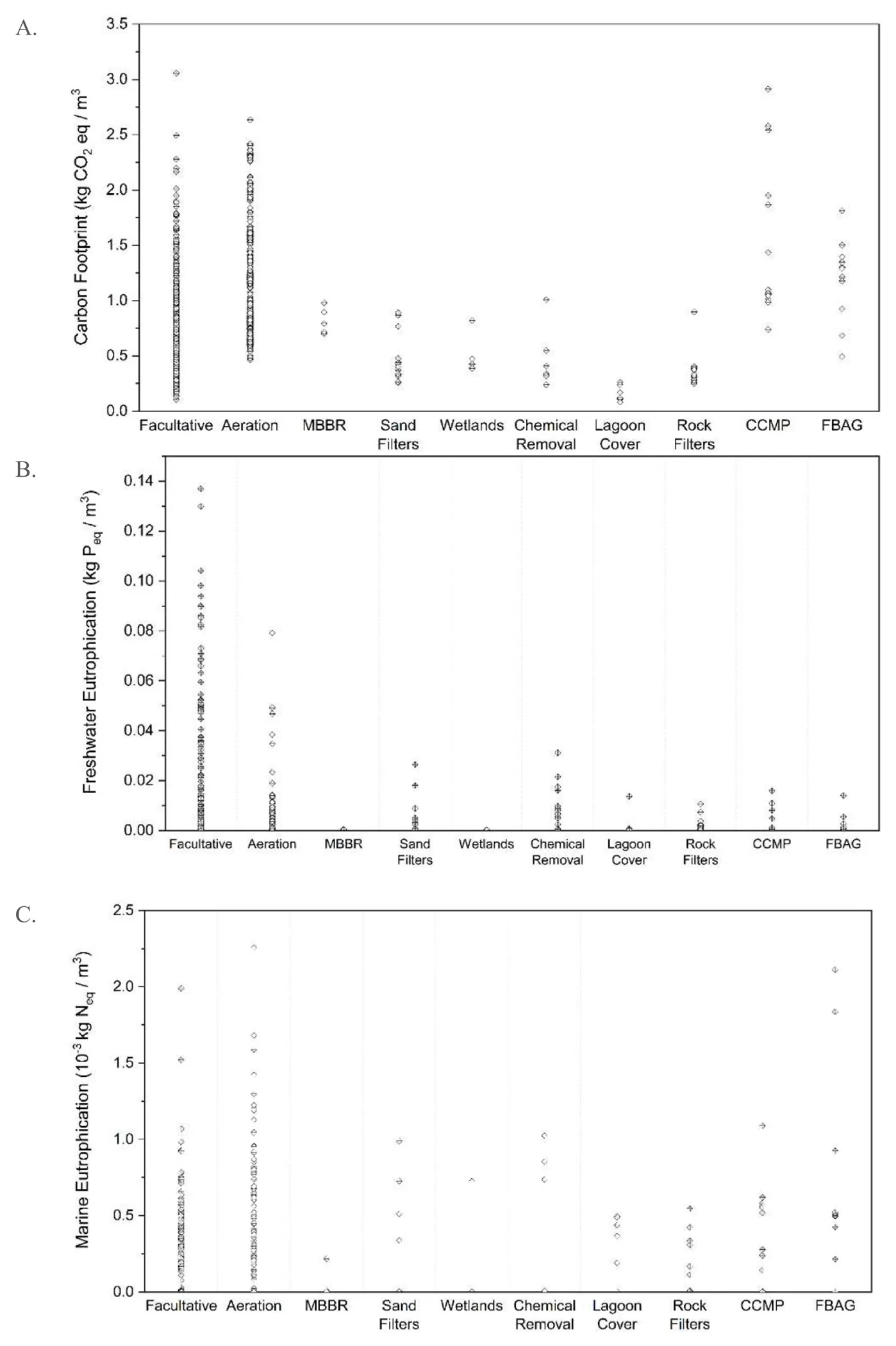
Environmental impact of nutrient management infrastructure in small community lagoon wastewater system. Each dot represents a facility. MBBR: moving -bed biofilm reactor; CCMP: covered complete-mix lagoon with polishing and FBAG: fixed-bed attached growth reactor.

Among all lagoons identified during the study (*n* = 4,678), the 3368 systems designated as facultative, including 2512 non-aerated facilities with available cell-level information, exhibited the highest carbon footprint (up to 3.1 kg CO_2_ eq m^−3^). This was attributed to methane and nitrous oxide emissions arising from anaerobic digestion in sludge layers and long hydraulic retention times that favor greenhouse gas formation (figure 7(a)). Aeration and CCMP also showed elevated carbon footprints (0.5–2.7 and 0.7–3.1 kg CO_2_ eq m^−3^, respectively) mainly from the energy-intensive nature of aeration systems and associated indirect CO_2_ emissions from electricity use (56%–97% contribution). This is based on the assumption that aerated cells were completely mixed, thus high energy intensity and negligible methane production. [23, 42, 43]

However, it is important to note that while aerated lagoons are assumed to operate under predominantly aerobic, completely mixed conditions, reported to strongly suppress methanogenesis relative to facultative or anaerobic lagoon systems, low-level methane production may still occur in poorly designed or managed systems or under localized and transient anoxic conditions [5, 13, 23]. This assumption reflects an idealized operational condition and does not imply complete absence of methane production under all field conditions. While inclusion of low-level methane emissions from aerated lagoons would increase absolute carbon footprint estimates, such contributions are expected to be small relative to operational energy-related emissions and unlikely to alter the relative ranking between aerated lagoon configurations and higher-intensity nutrient removal technologies at the screening level considered in this study.

In contrast, wetlands, sand filters, and chemical removal units showed considerably lower carbon footprint values, stemming from passive or low-energy operations. MBBR maintained intermediate carbon footprint values (0.5–1.1 kg CO_2_ eq m^−3^), balancing energy demand with effective nutrient removal. Lagoon covers have a low carbon footprint because they are passive technology that does not include any additional energy requirement for operation. Notwithstanding the embedded carbon in their materials, they prevent potent greenhouse gases from escaping by trapping significant methane and nitrous oxide emissions.

The FEP was also highest for facultative (up to 0.14 kg P eq m^−3^) and aerated lagoons (up to 0.08 kg P eq m^−3^) (figure 7(b)). This was mainly attributed to elevated effluent phosphorus concentrations arising from the typically low phosphorus removal efficiency of these systems [2]. In contrast, chemical removal, wetlands, and biofilm-based technologies (MBBR and FBAG) exhibited much lower FEP values (maximum of 0.0005 and 0.014 kg P eq m^−3^, respectively) from improved phosphorus removal. This suggests that implementing such upgrades can substantially mitigate eutrophication risk in freshwater environments.

The MEP results followed a similar trend to FEP (figure 7(c)). Facultative and aerated lagoons again displayed the highest MEP values due to relatively high effluent TN concentrations as expected from these systems [30]. In contrast, facilities equipped with enhanced nutrient removal technologies such CCMP, FBAG and MBBR achieved substantially lower nitrogen-equivalent emissions, with MEP values below 0.005 kg N eq m^−3^; similarly reported in other studies [13, 44]. CWs also performed well, producing comparatively low marine eutrophication impacts (<0.0075 kg N eq m^−3^), reflecting their natural capacity for nitrogen uptake and transformation [41].

Notably, across all impact categories (carbon footprint, FEP and MEP), there was significant variability. This was attributed to differences in treatment mechanisms, energy demand, and sensitivity to environmental conditions. Systems relying heavily on biological processes, such as facultative and aerated lagoons, showed greater variability because their performance and associated emissions fluctuate with temperature, organic loading, and microbial activity. Warm seasons enhance nutrient removal and lower eutrophication potential, while cold conditions suppress nitrification and denitrification, increasing nitrogen and phosphorus discharges [1, 13]. Aerated systems also experience variability linked to aeration intensity and electricity-related emissions [26]. In contrast, specialized nutrient technologies such as MBBR, CCMP, FBAG, and chemical precipitation exhibit more stable environmental profiles due to stronger process control and lower temperature sensitivity, achieving consistent nutrient removal.

It is worth noting that the number of facilities contributing data to each technology category varied, leading to unequal sample sizes across treatment types. While this discrepancy does not alter the overall patterns observed in carbon footprint and eutrophication impacts, it provides important context for interpreting the spread of data points within each category.

### 3.6. Limitations

This study integrates multiple federal datasets that collectively provide a comprehensive national-scale information on nutrient management infrastructure in small community lagoon wastewater treatment systems, while also introducing important limitations that warrant explicit consideration. Systematically missing data constrained analysis, particularly for operational characteristics, nutrient performance metrics, and upgrade histories, which are inconsistently reported across federal databases. These data gaps necessitated exclusion of facilities lacking minimum reporting completeness, leading to reduced and variable sample sizes across individual analyses. Consequently, facility counts differ among datasets and analytical components, affecting statistical power and limiting direct comparability across results. These limitations underscore that the findings should be interpreted as indicative of national-scale patterns and relative trends rather than exhaustive characterization of all lagoon systems in the United States.

Since these databases rely primarily on permitting, compliance reporting, and voluntary survey responses, the analyzed facility cohort may be subject to selection bias, with greater representation of permitted, actively reporting, and discharging facilities. As a result, non-discharging lagoons, facilities with intermittent operation, or systems with incomplete regulatory records are likely underrepresented, despite their prevalence in small community contexts.

Additionally, influent concentration and mass loading data were largely unavailable in public databases. When facility-specific influent concentration data were not available, influent mass loadings were estimated using average per-capita loading rates reported in peer-reviewed literature and scaled by the population served. While this approach introduced uncertainty in absolute loading estimates, it was unlikely to affect relative comparisons across treatment configurations at the screening level considered in this study.

Direct greenhouse gas emissions were estimated using influent BOD as a simplified proxy for organic loading. This approach represents a screening-level approximation and does not explicitly capture the complex, site-specific processes governing greenhouse gas generation in lagoon systems. Methane and nitrous oxide emissions are strongly influenced by redox conditions, lagoon depth, hydraulic retention time, aeration status, temperature, and seasonal variability, which are not resolved using influent loading alone [26, 43, 45]. Additionally, uncertainty in influent BOD propagated to carbon footprint estimates, affecting absolute values; however, consistent application of these assumptions across technologies supported the robustness of relative comparisons. Accordingly, estimated direct emissions should be interpreted as relative indicators for comparative assessment rather than precise quantification of facility-specific greenhouse gas fluxes.

## 4. Conclusions

This study provides a comprehensive assessment of lagoon wastewater treatment systems in small U.S. communities, highlighting key treatment processes, regulatory context, nutrient management capacity, and environmental performance. The analysis revealed that most lagoon systems serve small populations with flows below 0.1 MGD, with limited nutrient removal capacity. Nutrient parameters, particularly TN and TP, remain inconsistently monitored and rarely subject to enforceable limits. This regulatory gap, coupled with the lack of standardized influent data collection practices, limits the ability to quantify nutrient loading and evaluate treatment efficiency across systems. Addressing these gaps is essential for advancing more accurate performance benchmarking for lagoon optimization.

In terms of treatment processes, non-aerated lagoons remained the predominant configuration, though an increasing number of systems have incorporated aerated cells, lagoon modifications, addon, and post-treatment processes to enhance BOD and ammonia removal. Nonetheless, there is still a persistent shortfall in nutrient management capacity. Targeted retrofits such as biofilm reactors and gravel-bed filters have shown superior nutrient removal and more stable year-round performance, particularly for ammonia and TN, while chemical precipitation remains the most effective option for phosphorus control. These findings suggest that incremental, add-on technologies, rather than wholesale system replacement, represent a practical and scalable pathway for improving nutrient performance in small community lagoons.

Environmental impact analysis further demonstrated that facultative and aerated lagoons without nutrient removal exhibit higher eutrophication and carbon footprints relative to systems incorporating nutrient control technologies. Importantly, several upgrade options achieved improved nutrient removal without disproportionate increases in greenhouse gas emissions, indicating that nutrient and climate objectives need not be treated as competing priorities. Accordingly, lagoon upgrade decisions should prioritize technologies that deliver demonstrable nutrient reductions while maintaining low operational energy demand, particularly in communities with limited financial and operational capacity.

Taken together, these findings yield several actionable implications. For regulators, the results support prioritizing standardized nutrient monitoring requirements and incorporating nutrient performance metrics into lagoon permitting frameworks. For utilities and technical assistance providers, the study highlights retrofit-based nutrient management strategies as viable, lower-risk interventions that can substantially improve performance without requiring complete system replacement. For planners and funding agencies, integrating life cycle environmental and cost considerations into early-stage decision-making can help align infrastructure investments with both water quality and climate goals.

Potential areas for future research include leveraging the vast nutrient datasets generated through routine monitoring to enhance compliance oversight and support data-driven nutrient regulation. Establishing a national nutrient discharge database with standardized influent and effluent reporting would provide a stronger foundation for comparative assessment and policy development. Future work could also focus on quantifying greenhouse gas emissions from individual lagoon unit processes and applying data-driven modeling and remote sensing to map system performance and emissions at scale. Collectively, addressing these gaps will strengthen regulatory consistency, guide context-specific and cost-effective upgrades, and advance the transition toward nutrient-smart, climate-resilient small community lagoon wastewater systems.

## Supplementary Material

Supplementary Tables

Supplementary material for this article is available online

## Figures and Tables

**Table 1.** Influent characteristics based on monthly average and 30 d average data. BOD: biochemical oxygen demand; cBOD-carbonaceous BOD; NO2 + NO3: nitrite and nitrate; TKN: total kjeldahl nitrogen; TN: total nitrogen; TP: total phosphorus; TSS: total suspended solids.

Parameter	Units	№ of facilities with parameter	Data points	Mean	Median	Standard deviation
BOD	mg l^−1^	1527	475 000	158	130	123
cBOD	mg l^−1^	246	9312	170	136	138
TSS	mg l^−1^	1599	49 930	146	98.5	140
NH_3_	mg l^−1^	67	995	25.2	22.3	17.0
NO_2_/NO_3_	mg l^−1^	55	535	2.32	0.35	9.02
TKN	mg l^−1^	114	2386	34.3	32.9	18.3
TN	mg l^−1^	35	871	35.4	33.0	16.2
TP	mg l^−1^	110	2639	5.01	4.50	3.76

**Table 2.** Distribution of facilities with permitted and monitored parameters.

Parameter	Total facilities	Permitted facilities	Monitored facilities	Permitted: monitored ratio	Data points
NH_3_	3035	1442	1593	47:52	249 900
NO_3_/NO_2_	709	26	683	3:96	42 889
TKN	613	36	577	5:94	41 433
TN	800	63	737	7:92	42 969
TP	1518	217	1301	14:85	99 231
TSS	3966	3848	118	97:2	586 135

**Table 3.** Effluent quality based on monthly average and 30 d average data. BOD/cBOD: (carbonaceous) biochemical oxygen demand; NH_3_: ammonia; NO_3_/NO_2_: nitrate/nitrite; TKN: total kjeldahl nitrogen; TN: total nitrogen; TP: total phosphorus; TSS: total suspended solids.

Parameter	Facilities	Data points	Arith. mean	Geo. mean	Median	Standard deviation	5th percentile	95th percentile
Ammonia	1762	37 371	5.33	1.48	1.7	8.75	0.07	23.5
BOD/cBOD	2950	69 261	13.1	8.69	9.1	20.2	2.00	33.0
NO_2_ + NO_3_	435	8122	3.79	1.03	1.0	12.1	0.05	14.4
TKN	363	7834	8.19	5.03	5.82	8.26	0.71	24.6
TN	381	5916	10.8	7.78	8.7	8.79	1.75	26.8
TP	981	18 333	2.40	1.62	1.9	2.36	0.26	5.99
TSS	2971	70 184	20.7	12.8	14	20.4	2.00	62.0

**Table 4.** Nutrient management infrastructure currently implemented in small community lagoons and the corresponding expected performance.

Treatment stage	Unit process	Upgrade/retrofit goal	Facilities
Secondary	Aeration	Oxygen supply and mixing	1697
Secondary	Baffles	Improved hydraulics	202
Secondary	Recirculation	Process optimization	2
Secondary	Floating plants	Phytoremediation	2
Secondary	Chemical precipitation	Phosphorus removal	15
Secondary	Covered complete mix	Nitrification	49
Add-on	Fixed bed attached growth	Nitrification	38
Add-on	Moving bed biofilm reactor	Nitrification	23
Post-treatment	Land application	Water reuse	12
Post-treatment	Rapid sand filter	Algae & suspended solids filtration	20
Post-treatment	Slow sand filter	Algae & suspended solids filtration	60
Post-treatment	Mixed-media filter	Algae & suspended solids filtration	2
Post-treatment	Rock filter	Algae & suspended solids filtration	31
Post-treatment	Intermittent sand filters	Algae & suspended solids filtration	70
Post-treatment	Wetland	Nitrogen and phosphorus removal	13

**Table 5.** Lagoon technologies and corresponding average effluent concentrations. FBAG: fixed-bed aerated gravel filters; CCMP: covered complete mix lagoon with polishing; MBBR: moving-bed biofilm reactor.

Technology	Ammonia		Total nitrogen		Total phosphorus	
	Lagoon facilities	NH_3 effluent_ (mgl^−1^)	Lagoon facilities	TN _effluent_ (mgl^−1^)	Lagoon facilities	TP _effluent_ (mgl^−1^)
Aeration	369	0.03–9.77	17	0.52–21.8	30	0.05–6.67
Chemical precipitation	12	0.48–5.90	—	—	15	0.16–2.85
Facultative lagoon	473	0.04–13.9	1	11.1	29	0.18–6.18
Lagoon cover	11	0.90–5.02	—	—	1	3.14
CCMP	25	0.05–0.19	2	6.99–23.9	—	—
MBBR	12	0.87–0.87	1	8.95	—	—
Rock filter	17	0.08–3.19	—	—	2	0.83–1.55
Fixed bed attached growth	36	0.06–0.40	6	2.13–15.8	4	0.51–3.02
Sand filter	26	0.05–0.08	—	—	2	0.75–1.29
Constructed wetland	12	0.10–24.8	1	6.56	—	—

**Table 6.** Corresponding average compliance rate for ammonia, total nitrogen and total phosphorus. FBAG: fixed-bed aerated gravel filters; CCMP: covered complete mix lagoon with polishing; MBBR: moving-bed biofilm reactor.

Technology	Ammonia (<5 mg l^−1^)		Total nitrogen (<10 mg l^−1^)		Total phosphorus (<1 mg l^−1^)	
	Lagoon facilities	Compliance rate (average)	Lagoon facilities	Compliance rate (average)	Lagoon facilities	Compliance rate (average)
Aeration	369	53%	17	99%	30	90%
Chemical	12	80%	—	—	15	88%
precipitation						
Facultative lagoon	473	53%	1	99%	29	91%
Lagoon cover	11	63%	—	—	1	99%
CCMP	25	86%	2	99%	—	—
MBBR	12	78%	1	99%	—	—
Rock filter	17	65%	—	—	2	91%
Fixed bed	36	87%	6	99%	4	99%
attached Growth						
Sand filter	26	59%	—	—	2	98%
Constructed	12	71%	1	99%	—	—
wetland						

## Data Availability

All data that support the findings of this study are included within the article (and any supplementary information files). Supplementary Tables available at https://doi.org/10.1088/3033-4942/ae5e00/data1.
